# Incidence of central retinal artery occlusion peaks in winter season

**DOI:** 10.3389/fneur.2024.1342491

**Published:** 2024-01-22

**Authors:** Caroline J. Gassel, Wolfgang Andris, Sven Poli, Karl Ulrich Bartz-Schmidt, Spyridon Dimopoulos, Daniel A. Wenzel

**Affiliations:** ^1^University Eye Hospital, Centre for Ophthalmology, University Hospital Tübingen, Tübingen, Germany; ^2^Department of Neurology and Stroke, University Hospital Tübingen, Tübingen, Germany; ^3^Hertie Institute for Clinical Brain Research, University Hospital Tübingen, Tübingen, Germany

**Keywords:** central retinal artery occlusion, stroke, incidence, seasonality, air pollution, public health, risk factors

## Abstract

**Introduction:**

Stroke incidence exhibits seasonal trends, with the highest occurrences observed during winter. This study investigates the incidence of central retinal artery occlusion (CRAO), a stroke equivalent of the retina, and explores its monthly and seasonal variations, as well as potential associations with weather and ambient air pollutants.

**Methods:**

A retrospective search of medical records spanning 15 years (January 2008–December 2022) was conducted at the University Eye Hospital Tübingen, Germany, focusing on diagnosed cases of CRAO. Incidences were evaluated on a monthly and seasonal basis (winter, spring, summer, fall). Weather data (temperature, precipitation, atmospheric pressure) and concentrations of ambient air pollutants [fine particulate matter (PM2.5), coarse particulate matter (PM10), nitrogen dioxide (NO_2_), and ozone (O_3_)], were analyzed for a potential association with CRAO incidence.

**Results:**

Out of 432 patients diagnosed with CRAO between 2008 and 2022, significantly varying incidences were observed monthly (*p* = 0.025) and seasonally (*p* = 0.008). The highest rates were recorded in February and winter, with the lowest rates in June and summer. Concentrations of NO_2_, PM2.5 and lower ambient air temperature (average, minimum, maximum) showed significant correlations with CRAO incidence.

**Discussion:**

This comprehensive 15-year analysis reveals a pronounced winter peak in CRAO incidence, with the lowest occurrences in summer. Potential associations between CRAO incidence and ambient air pollutants and temperature underscore the importance of considering seasonal trends and call for further investigations to elucidate contributing factors, potentially leading to targeted preventive strategies and public health interventions.

## Introduction

Central retinal artery occlusion (CRAO) causes acute monocular vision loss and is a rare vascular occlusive event with an incidence of ~1.3–1.8 per 100,000 person-years (1, 2). CRAO is usually caused by thromboembolic occlusion of the central retinal artery and shares etiologic and risk factors with other ischemic vascular disorders, such as ischemic cerebral stroke, which is why CRAO is considered a stroke equivalent (3). CRAO patients are at high risk of suffering simultaneous cerebral stroke, which is frequently documented in close temporal proximity (4–9). Endogenous vascular risk factors of CRAO are well-known, of which the most prevalent are arterial hypertension, carotid stenosis, atrial fibrillation, and valvular heart disease (5, 7, 10, 11). However, numerous studies have reported a seasonal variation in the incidence of ischemic cerebral stroke with an increased number of patients suffering from stroke in winter and lower incidence in summer, suggesting a significant impact of external risk factors, such as ambient air pollution or temperature (12–20). Significant temperature fluctuations and exposure to air pollutants are increasingly recognized as important exogenous environmental risk factors for ischemic events. Despite their shared etiology and clinical correlations, it is unclear if the relationship between ischemic cerebral stroke and seasonal patterns, climatic influences, and air pollution levels also exists for CRAO. Therefore, this study seeks to investigate whether there are monthly or seasonal variations in the incidence of CRAO and probes potential associations with specific seasonal variations, focusing on weather parameters and ambient air pollutants. Such findings may hold substantial significance in guiding public health interventions.

## Materials and methods

### Study design and data collection

A retrospective analysis of medical records was conducted at the University Eye Hospital Tübingen to investigate the incidence of non-arteritic CRAO from January 2008 to December 2022. Newly diagnosed CRAO cases during this period were identified, and incidence dates were recorded. Patients showing signs of arteritic CRAO, based on their medical history, clinical symptoms (like headache, jaw claudication, and scalp tenderness), and/or increased inflammatory markers (such as elevated erythrocyte sedimentation rate or c-reactive protein), were excluded from the cases. Cumulative monthly (January–December) and seasonal incidence rates (number of patients that presented with CRAO during the given period) were calculated. For the seasonal analysis, the number of events was aggregated across four seasons and defined as winter (December–February), spring (March–May), summer (June–August), and fall (September–November). Normalization was performed to account for variations in month lengths.

### Weather and air pollution analysis

A comprehensive analysis of weather data and ambient air pollutants was conducted in the region surrounding the University Eye Hospital Tübingen, located in southwestern Germany. This area shows a combination of semi-urban and rural characteristics with mid-sized cities and experiences a temperate climate with warm summers and cold temperatures just above freezing to a few degrees below with occasional snowfall and frost during winter. The average annual temperature is around 10°C, with moderate temperature ranges throughout the year. Summers are mild to warm with the highest temperatures in July [mean average temperature (*T*_avg_) during the study period: 19.82°C; mean minimum temperature (*T*_min_): 13.99°C; mean maximum temperature (*T*_max_): 25.38°C], while winters are cool, with January being the coldest month (mean *T*_avg_: 1.44°C; mean *T*_min_: −1.84°C; mean *T*_max_: 4.52°C). The region receives ~740 mm of annual precipitation, distributed relatively evenly across the months with somewhat higher rainfalls in summer. Compared to some other regions in Germany, Tübingen tends to have slightly milder temperatures, especially during winter, owing to its more southerly location. In comparison to northern cities like Hamburg or Berlin, Tübingen generally enjoys somewhat warmer weather throughout the year. However, it is important to note that Germany's climate variations aren't extreme, and differences between regions are often subtle. Weather data, including temperature, atmospheric pressure, and precipitation were obtained from meteostat.net (21), an open-source webpage providing weather and climate data from several (inter-)national meteorological governmental services, such as the “Deutscher Wetterdienst” (DWD). Ambient concentrations of fine particulate matter (PM2.5), coarse particulate matter (PM10), nitrogen dioxide (NO_2_), and ozone (O_3_) were considered as key air pollutants of interest. These pollutants are known to potentially have various adverse effects on human health and have been linked to cardiovascular and ocular diseases (18, 20, 22). Daily average measurement of ambient air pollutants (PM2.5, PM10, NO_2_, O_3_) were provided by the German Environment Agency (Umweltbundesamt).

### Statistical analysis

To assess potential variations in CRAO incidence across different months and seasons, we employed Chi-squared test. The null hypothesis assumed a homogenous distribution of the number of CRAO cases per month or season throughout the entire year. If statistically significant differences were found, it would suggest that the distribution of CRAO cases deviated from uniformity across months or seasons, indicating potential monthly or seasonal variations in CRAO incidence.

Spearman's correlation coefficients were used to explore relationships between daily pollutant concentrations and CRAO incidence. To address the challenge posed by the low annual incidence of CRAO events, we implemented two analytical approaches: a continuous analysis and a cumulative analysis. The continuous approach involved assessing CRAO incidence in relation to daily air pollutant concentrations, providing a day-to-day perspective. Conversely, the cumulative approach amalgamated data over the 15-year study duration, creating an averaged representation of pollutant concentrations (PM.25, PM10, NO_2_, O_3_) and weather variables for each day, simulating a hypothetical year. This cumulative methodology aimed to enhance the statistical power and sensitivity of our analysis, allowing us to detect possible correlations that might not be apparent when examining individual days, months or years with a low incidence of CRAO.

A *p*-value threshold of < 0.05 was chosen to denote statistically significant differences. All calculations were performed using Python (version 3.10).

### Ethics approval

This study adhered to the tenets of the Declaration of Helsinki. Ethical approval was obtained from the institutional ethics committee of the University Hospital Tübingen (project number: 584/2023BO2).

## Results

Between January 2008 and December 2022, a total of 432 patients with a mean age of 72.4 ± 12.1 years (mean ± SD), were newly diagnosed with CRAO. Among these, 254 patients (58.8%) were male and 178 patients (41.2%) were female (*p* = 0.0003). Two hundred forty cases (55.6%) involved the right eye, while 192 cases (44.4%) involved the left eye (*p* = 0.021).

**Figure 1:**
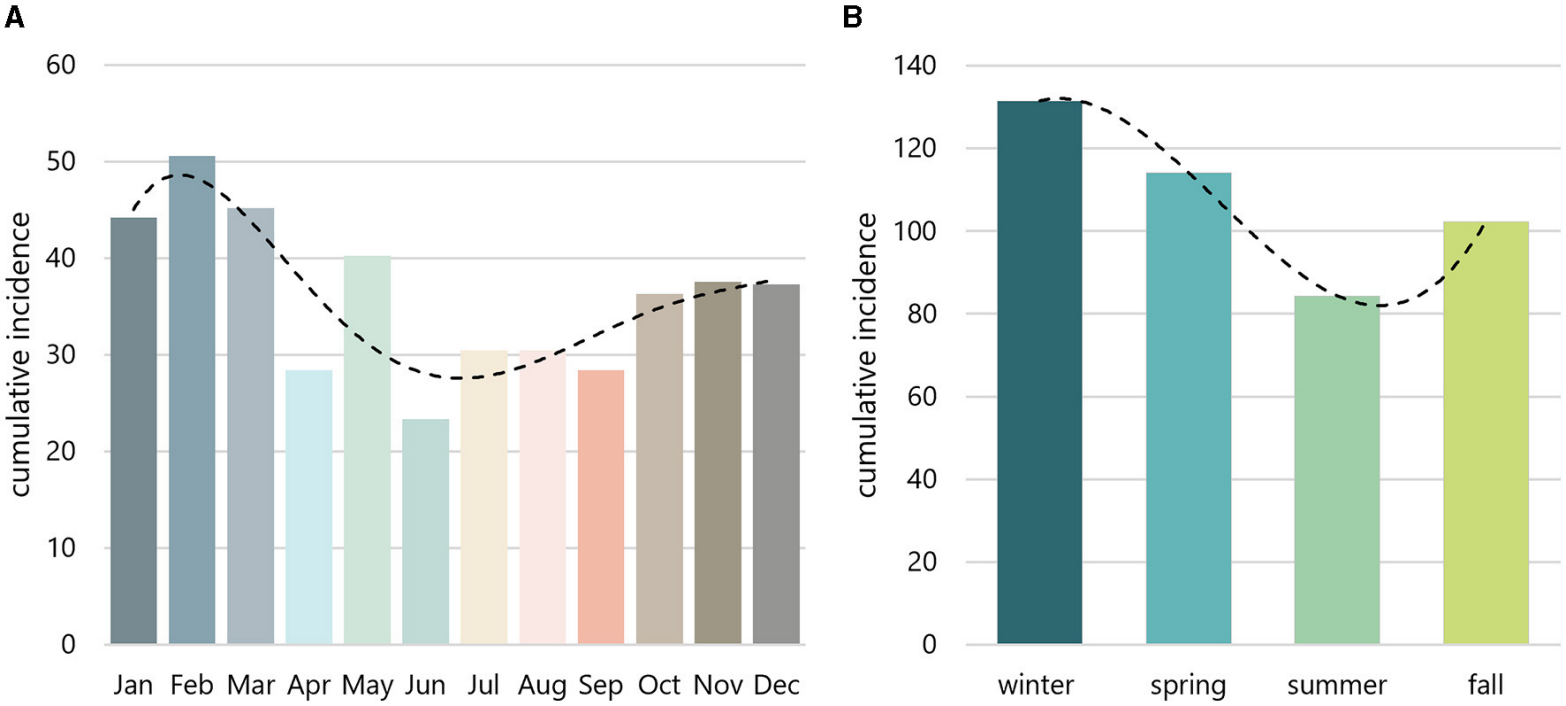
Monthly and seasonal incidence. Monthly **(A)** and seasonal **(B)** incidences were aggregated over a 15-year period and adjusted based on the varying lenghts of months. The peak incidence emerged in February **(A)** and winter **(B)**, whereas the nadir was observed in June **(A)** and summer **(B)**. Dashed line indicates trendline.

The CRAO incidence was compared across all 12 cumulative monthly and all four cumulative seasonal incidences. The cumulative monthly and season-specific adjusted incidence rates over the 15-year period are shown in Figure 1 and Table 1. Our data clearly demonstrate significant variations in the 15-year cumulative incidence rates across months (*p* = 0.025) and seasons (*p* = 0.008). Notably, winter and spring showed the highest incidences of CRAO at 131.51 and 114.15, respectively. In contrast, the summer and fall seasons showed lower cumulative incidences at 84.37 and 102.35. Accordingly, the incidence during winter was 1.55 times higher than during summer. February had the highest cumulative adjusted incidence (50.61), more than double (factor 2.17) than the incidence in June (23.34), which marked the month with the lowest incidence. Both months showed the highest deviation from the expected incidence in a hypothetical homogenous distribution throughout the year.

**Table 1:** Incidence of central retinal artery occlusion per month and season.

**Month**	**Season**	**Incidence/ month**	**Incidence/ season**
Dec	Winter	37.31	131.51
Jan		44.19	
Feb		50.61	
Mar	Spring	45.17	114.15
Apr		28.41	
May		40.26	
Jun	Summer	23.34	84.37
Jul		30.44	
Aug		30.44	
Sep	Fall	28.41	102.35
Oct		36.33	
Nov		37.54	
χ^2^-test		*p* = 0.025	*p* = 0.008

Adjusted cumulative incidences over the study period of 15 years per month and per season. The cumulative incidences were adjusted allowing for a comparative assessment independent of variations in the lengths of months or seasons. The Chi-squared test indicates the significance of differences in incidence among months and seasons, revealing statistical significance for both comparisons (p = 0.025 for months; p = 0.008 for seasons).

**Figure 2:**
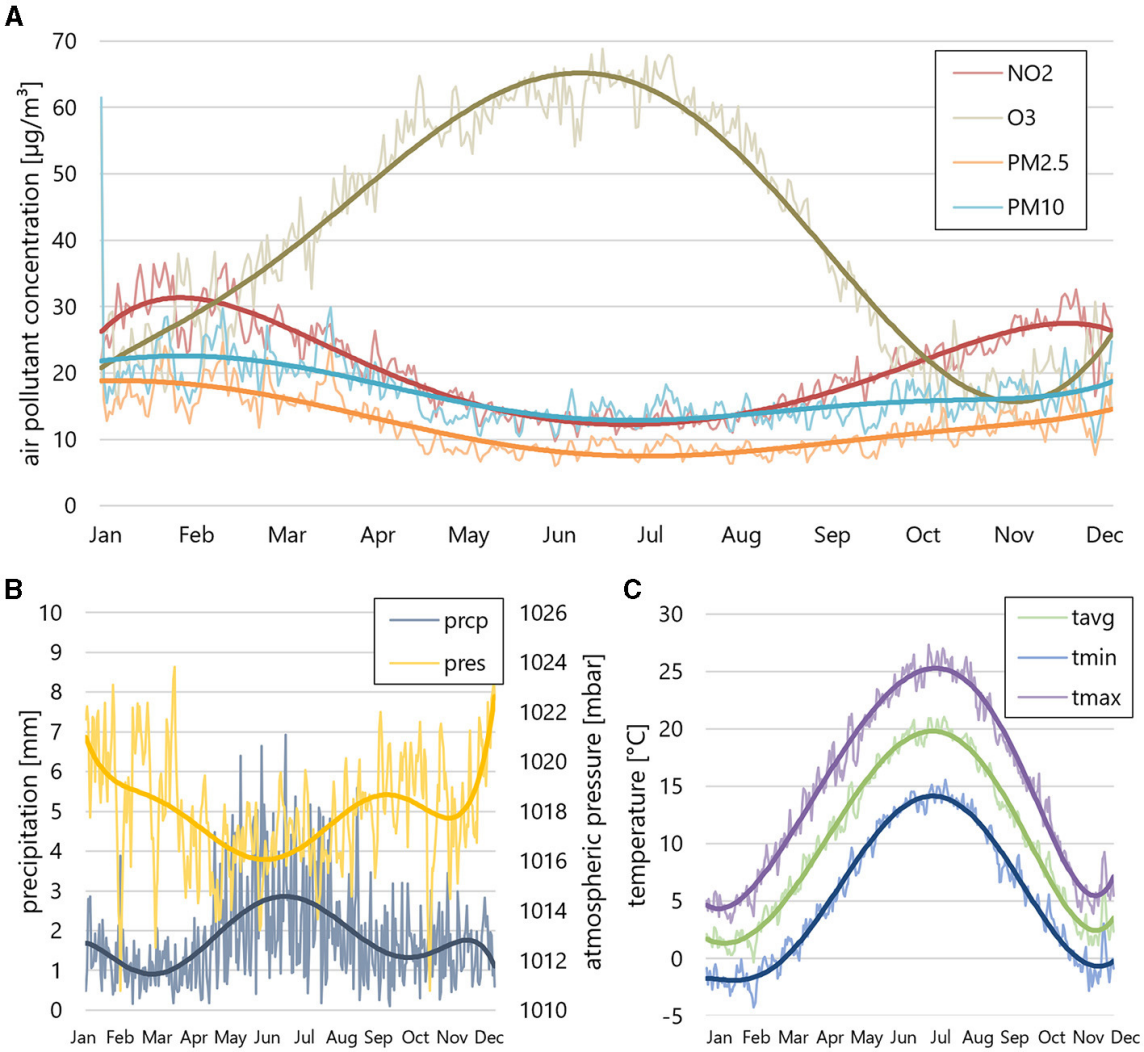
Air pollution and weather data. **(A)** Cumulative average air pollutions (NO_2_, O_3_, PM2.5, PM10), **(B)** precipitation (prcp) and atmospheric pressure (pres) and **(C)** mean temperature [mean average temperature (*T*_avg_), mean minimum temperature (*T*_min_), mean maximum temperature (*T*_max_)] during the 15-year study period. PM2.5, PM10, and NO_2_ show increased concentrations in the winter months, while O_3_ concentration peaks in summer. The mean temperature follows a seasonal pattern, with average temperatures between 0 and 5°C in winter and around 20° in summer.

The environmental factors, including weather and air pollution data are presented in Figure 2 and Table 2. Apart from O_3_, which peaks in summer and shows lower concentrations in winter, all other air pollutants showed increased concentrations in the winter months. Temperature trends followed the expected seasonal pattern, with average temperatures ranging from 0 to 5°C in winter and around 20°C in summer. A correlation analysis, conducted for both continuous and cumulative data, revealed a noteworthy inverse correlation between CRAO incidence and temperature. Specifically, mean *T*_avg_ [*p* = 0.018/0.004 (continuous/cumulative analysis)], mean *T*_min_ (*p* = 0.019/0.004) and mean *T*_max_ (*p* = 0.0299/0.004) exhibited significant correlations with CRAO incidence. Correlation coefficients in the cumulative analysis were similar for *T*_max_ (−0.151), *T*_min_ (−0.149) and *T*_avg_ (−0.147). Our findings suggest a weak to moderate negative monotonic relationship between temperature and the continuous/cumulative incidences. Precipitation and atmospheric pressure did not show significant correlations.

**Table 2:** Cumulative mean concentrations of air pollutants and weather data.

**Month**	**NO_2_ (μg/m3)**	**O^3^ (μg/m3)**	**PM_2.5_ (μg/m3)**	**PM_10_ (μg/m3)**	***T*_avg_ (°C)**	***T*_min_ (°C)**	***T*_max_ (°C)**	**Prcp (mm)**	**Pres (mbar)**
Jan	30.28	24.17	18.41	22.14	1.44	−1.84	4.52	1.40	1,020.04
Feb	29.41	31.06	17.62	21.98	2.46	−1.56	6.63	1.10	1,019.26
Mar	25.71	41.25	16.94	22.22	5.94	0.80	11.03	1.05	1,018.27
Apr	20.07	53.78	12.03	17.75	10.19	4.30	15.66	1.42	1,016.19
May	14.73	58.77	8.46	13.17	14.00	8.33	19.23	2.43	1,016.72
Jun	12.86	62.07	8.43	13.48	18.16	12.54	23.46	3.12	1,016.62
Jul	12.84	63.75	8.74	14.56	19.82	13.99	25.38	2.59	1,016.72
Aug	13.03	55.98	8.09	13.75	19.19	13.55	24.87	2.17	1,017.16
Sep	17.27	40.06	8.74	13.97	14.81	9.46	20.34	1.31	1,018.74
Oct	21.09	23.07	10.56	15.62	10.21	5.57	15.23	1.59	1,019.24
Nov	24.09	17.66	12.13	16.16	5.45	1.83	9.20	1.58	1,017.10
Dec	27.57	20.30	13.23	17.07	2.66	−0.62	5.80	1.55	1,019.63

Data shown as mean cumulative values during the 15-year study period for each month.

Additionally, the correlation analysis between air pollutants and CRAO incidence uncovered a significant relationship between CRAO and NO_2_ concentrations [*p* = 0.011/0.008 (continuous/cumulative analysis)] as well as PM2.5 (*p* = 0.050/0.049). Other air pollutants (O_3_, PM10) did not exhibit such correlations.

**Figure 3:**
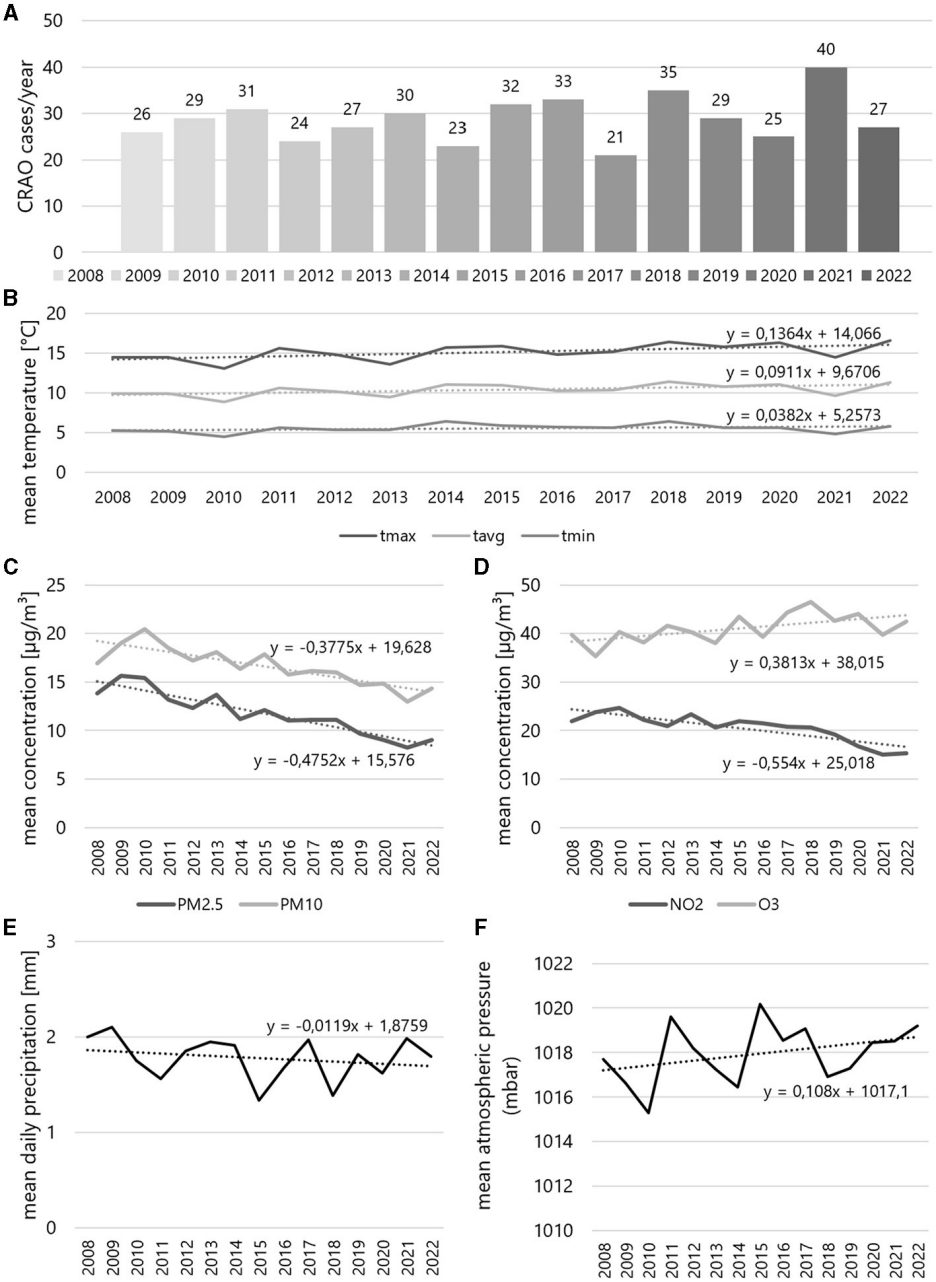
Yearly incidences of central retinal artery occlusion and corresponding yearly weather and air pollution data during study period. **(A)** Incidences by year from 2008 to 2022, yearly mean **(B)** temperature and concentrations of **(C)** PM2.5 and PM10, **(D)** NO_2_ and O_3_, and **(E)** mean daily precipitation and **(F)** mean atmospheric pressure. The formulas relate to the respective trendlines of the data curve.

The annual CRAO incidence fluctuated between 21 and 40 cases per year, remaining relatively stable over the observation period (see Figure 3). The average yearly temperature (for *T*_avg_, *T*_max_, *T*_min_) exhibited a slight upward trend over the 15 years, while air pollutant concentrations (PM2.5, PM10, NO3) tended to decrease, except for O3, which showed a slight increase (see Figure 3).

## Discussion

This study provides a comprehensive examination of the incidence of CRAO over a 15-year period, investigating previously unexplored monthly and seasonal variations in a region with mostly mid-sized cities in a temperate climate zone. The observed peak in CRAO incidence during winter aligns with patterns observed in ischemic vascular diseases and ischemic cerebral strokes, hinting shared risk factors and a potential link to environmental factors (12–17, 19, 23). Previous studies have established a connection between cold temperatures as well as air pollution with an elevated risk of ischemic cerebral stroke. This association is attributed to the potential impact of lower temperatures and increased air pollution on factors like blood pressure, hemorheology, and clotting factors (24–28). Our findings for CRAO align with previously published data, showcasing a correlation between colder temperatures, higher air pollutant concentrations and increased risk of cerebral ischemic events (19, 27, 29–32).

Air pollution includes airborne particulate matter (PM) such as PM10 and PM2.5, and gases like NO_2_, O_3_, carbon monoxide (CO), and sulfur dioxide (SO_2_). PM2.5, which is generated from fossil fuel combustion and is particularly hazardous due to its small size, can infiltrate the respiratory system, as well as enter the blood stream. PM10 originates from road dust, industry, and pollen mainly affecting the respiratory system (33). NO_2_, a combustion byproduct also mainly irritates the respiratory system. Ground-level O_3_, formed by nitric oxides and volatile organic compounds reacting in sunlight, is both protective high up in the atmosphere (stratospheric O_3_) and harmful at ground level, causing mainly respiratory issues. Vehicle and industrial emissions are major contributors to O_3_ precursor pollutants.

The relevance of ambient air pollution has grown significantly due to its role as a major risk factor for vascular events like stroke and also overall mortality (20, 34–39). A comprehensive meta-analysis covering 6.2 million ischemic events across 28 countries found a significant association between increased concentrations of NO2, SO2, CO, PM2.5, and PM10 and the relative risk of stroke incidence or mortality (36). The increased levels of pollutants in the winter, including NO_2_ and particulate matter (PM2.5 and PM10), correspond to the seasonal patterns in CRAO occurrence with a higher number of CRAO cases in winter. Interestingly, the incidence was the highest in the months with the highest concentrations of PM2.5 and NO_2_. Higher NO_2_ and PM2.5 levels during colder months might exacerbate vascular risk factors, correlating with the increased CRAO incidence.

Interestingly, a Taiwanese study from 2016, also reported a correlation of ambient air pollution and the incidence of CRAO (18). In that study, a retrospective analysis of patients newly diagnosed with CRAO between 2001 and 2013 based on the Taiwan National Health Insurance Research Database was conducted. More than other pollutants, an increase in NO_2_ and SO_2_ increased the risk of CRAO, while PM2.5, PM10, and O_3_ did not have significant effects, an observation that at least partially aligns with our observations. However, only 96 patients were examined in this study, significantly limiting the conclusiveness regarding causality. Therefore, based on current evidence, it has not been sufficiently established whether air pollution is indeed a risk factor for CRAO. Another study from Poland found that short-term exposure to several air pollutants (NO_2_, SO_2_, O_3_, CO, and PM10) might be related to CRAO (40). The higher levels of air pollution in winter are not confined to Tübingen, Germany; they are a common trait in colder regions or seasons (41–44). Cold air tends to stay close to the ground, making it harder for pollutants to disperse freely. Additionally, colder air lacks the humidity needed to effectively clear pollutants. With winter's increased energy usage for heating and more fossil fuel combustion, pollution levels tend to rise. Grzybowski and Mimier's (40) study supports this, noting a surge in pollution-related incidents following a drop in air temperature, emphasizing the influence of weather on air quality. Interestingly, we found a correlation of CRAO incidence with PM2.5 but not PM10 in our study, although both concentrations were almost parallel in the course of the cumulative analysis. As mentioned, PM2.5 is known to be more relevant for vascular occlusive events, as PM10 is considered to only penetrate the respiratory system, whereas PM2.5 can enter the blood system, where it may exert proinflammatory and prothrombogenic effects. This might explain the stronger correlation between CRAO incidence and PM2.5 in contrast to PM10 (33, 45, 46).

This study aligns with a growing body of evidence suggesting the potential influence of seasonal variations, climatic factors, and air pollution on vascular events. Notably, it marks the first instance of demonstrating seasonal fluctuations in CRAO incidence. However, further exploration is necessary to fully grasp the mechanisms connecting these seasonal shifts with CRAO occurrences. Understanding these mechanisms could pave the way for targeted public health interventions, particularly during high-incidence seasons, mitigating CRAO risks related to air pollution. Additionally, emphasizing these findings within the climate emergency framework accentuates the significance, highlighting the potential exacerbation of ocular vascular risks in worsening environmental conditions. Yet, the study has limitations that warrant consideration. Relying on available medical records in a retrospective design might lead to underreporting or incomplete data, potentially skewing the findings. Additionally, the study's singular institution focus limits generalizability, urging future research across diverse populations and regions with varying climate characteristics. Bearing in mind that the study's site (Tübingen) is not a city with extremely high levels of air pollution, more extensive studies should be carried out in cities with higher concentrations of air pollutants or in regions with less pollution. The study acknowledges limitations in analyzing all air pollutants due to data constraints, indicating the need for comprehensive evaluations in subsequent studies. Moreover, considering lifestyle factors and comorbidities not accounted for in this study requires prospective investigations to minimize biases associated with retrospective analyses. Despite these limitations, the study's strengths lie in its extensive 15-year retrospective analysis, multi-dimensional approach, and identification of seasonal patterns, laying a foundation for understanding environmental risk factors. Future research involving larger datasets and comparative studies across varied geographic settings could further illuminate the impact of weather and air pollution on CRAO incidence, contributing to a better understanding of vascular diseases' risk factors in the broader context.

In conclusion, this comprehensive 15-year analysis reveals evidence of seasonal and monthly variations in CRAO incidence, with a pronounced peak during winter. Moreover, a potential association between CRAO incidence and air pollutants and lower temperature was found. These findings prompt further research into the underlying mechanisms and potential modifiable risk factors associated with these temporal patterns. Such investigations could ultimately lead to more effective and targeted preventive strategies to reduce the risk of CRAO.

## Data availability statement

The raw data supporting the conclusions of this article will be made available by the authors, without undue reservation.

## Ethics statement

The studies involving humans were approved by the Ethics Committee at the Faculty of Medicine of the Eberhard Karls University and at the University Hospital Tübingen. The studies were conducted in accordance with the local legislation and institutional requirements. The Ethics Committee/institutional review board waived the requirement of written informed consent for participation from the participants or the participants' legal guardians/next of kin because, due to the study's retrospective nature and lengthy inclusion period, obtaining individual consent from each patient was deemed impractical. The overall benefit of the research outweighs the need for individual consent as the findings could impact future health outcomes. The study follows ethical guidelines and data protection laws, ensuring strict confidentiality and data anonymization to safeguard patient identities. Importantly, the study's implementation does not affect the ongoing medical care of the participants.

## Author contributions

CG: Formal analysis, Methodology, Validation, Writing—original draft, Writing—review & editing, Conceptualization, Data curation, Investigation. WA: Conceptualization, Data curation, Formal analysis, Investigation, Methodology, Validation, Writing—review & editing. SP: Methodology, Validation, Writing—review & editing. KB-S: Methodology, Supervision, Validation, Writing—review & editing. SD: Formal analysis, Methodology, Supervision, Validation, Writing—review & editing. DW: Conceptualization, Data curation, Formal analysis, Investigation, Methodology, Project administration, Supervision, Validation, Visualization, Writing—original draft, Writing—review & editing.
